# Early Integrated Palliative Care Within a Surgical Oncology Clinic

**DOI:** 10.1001/jamanetworkopen.2023.41928

**Published:** 2023-11-07

**Authors:** Varun V. Bansal, Daniel Kim, Biren Reddy, Hunter D. D. Witmer, Ankit Dhiman, Frederick A. Godley, Cecilia T. Ong, Sandra Clark, Leah Ulrich, Blase Polite, Ardaman Shergill, Monica Malec, Oliver S. Eng, Sandy Tun, Kiran K. Turaga

**Affiliations:** 1 Division of Surgical Oncology, Yale School of Medicine, New Haven, Connecticut; 2 Pritzker School of Medicine, University of Chicago Medical Center, Chicago, Illinois; 3 Department of Surgery, Division of General Surgery and Surgical Oncology, University of Chicago Medical Center, Chicago, Illinois; 4 Department of Surgery, Medical College of Georgia, Augusta; 5 Department of Medicine, Section of Hematology and Oncology, University of Chicago Medical Center, Chicago, Illinois; 6 Department of Medicine, Section of Geriatrics and Palliative Medicine, University of Chicago Medical Center, Chicago, Illinois; 7 Department of Surgery, Division of Surgical Oncology, University of California, Irvine

## Abstract

**Importance:**

Advance directive (AD) designation is an important component of advance care planning (ACP) that helps align care with patient goals. However, it is underutilized in high-risk surgical patients with cancer, and multiple barriers contribute to the low AD designation rates in this population.

**Objective:**

To assess the association of early palliative care integration with changes in AD designation among patients with cancer who underwent surgery.

**Design, Setting, and Participants:**

This cohort study was a retrospective analysis of a prospectively maintained registry of adult patients who underwent elective surgery for advanced abdominal and soft tissue malignant tumors at a surgical oncology clinic in a comprehensive cancer center with expertise in regional therapeutics between June 2016 and May 2022, with a median (IQR) postoperative follow-up duration of 27 (15-43) months. Data analysis was conducted from December 2022 to April 2023.

**Exposure:**

Integration of ACP recommendations and early palliative care consultations into the surgical workflow in 2020 using electronic health records (EHR), preoperative checklists, and resident education.

**Main Outcomes and Measures:**

The primary outcomes were AD designation and documentation. Multivariable logistic regression was performed to assess factors associated with AD designation and documentation.

**Results:**

Among the 326 patients (median [IQR] age 59 [51-67] years; 189 female patients [58.0%]; 243 non-Hispanic White patients [77.9%]) who underwent surgery, 254 patients (77.9%) designated ADs. The designation rate increased from 72.0% (131 of 182 patients) before workflow integration to 85.4% (123 of 144 patients) after workflow integration in 2020 (*P* = .004). The AD documentation rate did not increase significantly after workflow integration in 2020 (48.9% [89 of 182] ADs documented vs 56.3% [81 of 144] ADs documented; *P* = .19). AD designation was associated with palliative care consultation (odds ratio [OR], 41.48; 95% CI, 9.59-179.43; *P* < .001), palliative-intent treatment (OR, 5.12; 95% CI, 1.32-19.89; *P* = .02), highest age quartile (OR, 3.79; 95% CI, 1.32-10.89; *P* = .01), and workflow integration (OR, 2.05; 95% CI, 1.01-4.18; *P* = .048). Patients who self-identified as a race or ethnicity other than non-Hispanic White were less likely to have designated ADs (OR, 0.36; 95% CI, 0.17-0.76; *P* = .008). AD documentation was associated with palliative care consulation (OR, 4.17; 95% CI, 2.57- 6.77; P < .001) and the highest age quartile (OR, 2.41; 95% CI, 1.21-4.79; P = .01).

**Conclusions and Relevance:**

An integrated ACP initiative was associated with increased AD designation rates among patients with advanced cancer who underwent surgery. These findings demonstrate the feasibility and importance of modifying clinical pathways, integrating EHR-based interventions, and cohabiting palliative care physicians in the surgical workflow for patients with advanced care.

## Key Points

**Question:**

Is palliative care and surgical workflow integration associated with increased advance directive (AD) designation and documentation among patients with cancer undergoing surgery?

**Findings:**

In this cohort study of 326 patients with advanced abdominal and soft tissue malignant tumors evaluated in a surgical oncology clinic, palliative care consultation and workflow integration were associated with significantly increased rates of AD designation; the increase in AD documentation was not significant.

**Meaning:**

The findings of this study suggest that early palliative care integration in a surgical oncology service is feasible and may help promote timely advance care planning in this high-risk population.

## Teaser

This cohort study analyzes early palliative care integration and its association with changes in advance directive designations among patients with cancer who underwent surgery.

## Introduction

Effective palliative care in oncology enhances patient and caregiver experiences by improving symptom control, satisfaction, and quality of life during treatment.^1,2,3,4^ Additionally, it helps promote goal-concordant, end-of-life care through advance care planning (ACP).^4,5^ Although palliative care interventions mainly target the nonsurgical population, there is limited evidence regarding the benefits of these interventions (eg, facilitating decision-making and reducing health care resource utilization) for surgical candidates.^6,7,8,9,10^ Formalized incorporation of ACP in surgical patients remains historically low. Over two-thirds of patients undergoing high-risk surgery do not have designated advance directives (AD), and targeted interventions involving surgeon–palliative care comanagement for patients with advanced cancer remain scarce.^11,12,13^

Joint guidelines from the American College of Surgeons and the American Geriatric Society recommend AD designation as an important quality metric for ACP in surgical patients.^14,15^ However, barriers to ACP discussions contribute to suboptimal AD designation in the surgical oncology setting.^16^ Patients often lack awareness of these legal documents, avoid discussing death early in the treatment course, and tend to designate ADs during periods of clinical decline.^17,18,19^ Administrative hurdles to documentation include the lack of standardized systems for reporting and updating ADs.^20,21^ Surgeons may hesitate to facilitate ACP discussions due to time constraints or concerns that AD designation may limit treatment options they consider appropriate.^16,22,23,24,25^ Although ACP is associated with less aggressive and less expensive end-of-life care,^5,26^ health systems bear the cost of developing and delivering ACP interventions.^27,28^

Surgical encounters involve establishing substantial trust between stakeholders, presenting an ideal opportunity to facilitate ACP discussions.^12^ This intervention point is particularly important among patients with advanced cancer who are vulnerable to complications from surgery and the complexity of their disease. To address the aforementioned challenges, we implemented a novel initiative in our surgical oncology clinic, integrating ACP discussions throughout critical time points in the perioperative period for high-risk patients. We encouraged patients to discuss their goals of care with palliative and surgical teams augmented by oversight from a patient care coordinator. We hypothesized that palliative and surgical workflow integration would be associated with increased rates of AD designation. This study investigates the merits of the initiative, identifies its limitations, and provides recommendations for systematic changes required to enhance AD designation.

## Methods

### Study Design and Population

This cohort study followed the Strengthening the Reporting of Observational Studies in Epidemiology (STROBE) reporting guideline^29^ for observational studies and was approved by the University of Chicago institutional review board. Informed consent was not obtained for patients before 2020 because this study was considered a secondary analysis of deidentified data in accordance with 45 CFR § 46. All patients after 2020 gave written informed consent for prospective collection of their clinical demographic data, and this was a secondary analysis of the data. We performed a retrospective analysis of a prospectively maintained database from the Regional Therapies Surgical Oncology Service. Adult patients with advanced abdominal and soft tissue malignant tumors who underwent diagnostic and therapeutic surgical interventions between June 2016 and May 2022 were included in the study.

### Interventions

The Regional Therapies Surgical Oncology Service at our center was established in 2016 and cares for patients with locoregionally advanced and metastatic abdominal and soft tissue malignant tumors. Considering the extensive needs and benefits of palliative care use in this patient population,^30,31^ we onboarded a palliative care team comprising fellowship-trained palliative care attendings (including authors S.T. and M.M.) and a full-time patient care coordinator (author S.C.) in late 2017. The team assisted in designating and documenting ADs (namely, a health care power of attorney [HCPOA] and a living will [LW] using a structured clinical interview format) (eAppendix 1 in Supplement 1). Treating physicians strongly encouraged patients to designate ADs during clinic visits and referred them to palliative care as needed.

In 2020, the program was enhanced by integrating palliative and surgical workflows (eFigure in Supplement 1). Before each new patient visit, the patient care coordinator shared AD documents along with other hospital-related forms and offered an optional integrated palliative care consultation to incoming patients. A smart phrase computer code was embedded in our electronic health record (EHR) note-writing software to prepopulate AD parameters during surgical clinic visits.^32,33^ The recommendation for HCPOA designation was added to preoperative checklists and resident education curricula to increase engagement by surgical trainees. The patient care coordinator periodically reminded patients without an AD designation to complete it before surgery. Finally, ACP activities were reviewed in weekly and quarterly audits.

Notably, the palliative care team used the same clinic space and received assistance from surgical clinic nurses on days when the surgeons were operating. Daily huddles facilitated communication and handoffs between teams. This cohabiting structure aimed to optimize the function of the palliative care team, who gradually increased their time allocation from their outpatient practice to the Regional Therapies service.

### Study Variables and Outcomes

Baseline demographic, clinical, and treatment-related information was abstracted from medical records for all included patients. Data regarding patient-reported race and ethnicity were collected because prior reports have identified racial disparities in AD designation, and we wished to examine this association within our cohort. Race and ethnicity categories were defined as described in the institutional medical record system and included Asian and Mideast Indian, Black or African American, Hispanic and Latino, Native Hawaiian or Other Pacific Islander, non-Hispanic White, more than 1 race, and decline to answer. Considering the overrepresentation of non-Hispanic White patients in our cohort, we categorized patients belonging to all other racial and ethnic categories as *other populations* to preserve statistical power for group comparisons.

The primary study outcome was increased AD designation and documentation rates and we aimed to ascertain whether they were associated with workflow integration (beyond 2020). Designation was defined by the identification of an HCPOA in any clinical note by palliative care, surgical team member, or external clinicians; or a legal AD document (HCPOA or LW) scanned into the EHR.^12,34^ In the absence of documentation, a prespecified set of key terms was used to locate AD designation in progress notes (eAppendix 2 in Supplement 1).^9^

Various secondary outcomes were assessed. AD designation rates before and after workflow integration were compared between subgroups with and without palliative care consultations to assess whether processes other than palliative care visits were cumulatively associated with AD designation. The duration between the earliest AD identification in the EHR and the date of index surgery (defined as the first surgery recorded in our service case logs for each patient) was compared before and after workflow integration. Lastly, overall survival (OS) was compared between patients with and without palliative care visits.^35,36^

### Statistical Analysis

Descriptive statistics were generated, and multivariable logistic regression was performed to assess factors associated with AD designation and documentation. Variables with a *P* < .20 in univariable analyses were included in a backward conditional multivariable regression model, excluding patients with missing values. A 2-tailed *P* <. 05 was considered significant for group comparisons.

In post hoc analysis, OS was compared on the basis of palliative care consultation among patients with commonly encountered primary tumor sites (appendix, colorectal, and peritoneal mesothelioma) who underwent therapeutic procedures. OS was defined from the date of index surgery within our service to last clinical follow up. To minimize selection bias favoring patients not requiring palliative care consultation,^36^ a 1:1 nearest-neighbor propensity score–matched analysis was performed with a match tolerance of 0.02.^37^ Matched variables included age, primary site, preoperative American Society of Anesthesiology score, and treatment intent (curative vs palliative). The patients were censored on the date of the last follow-up visit.

All statistical analyses were performed using SPSS statistical software version 29.0 (IBM). Data analysis occurred from December 2022 to April 2023.

## Results

**Figure 1.**
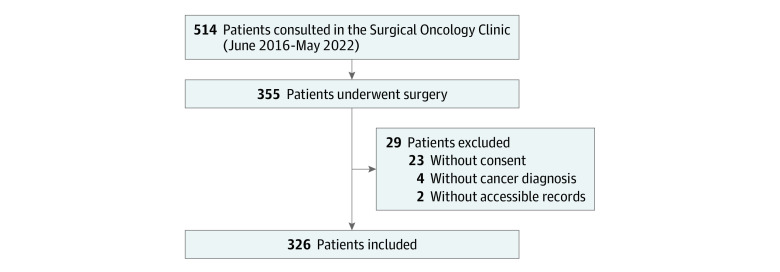
Flow Diagram for Patient Inclusion

The study included 326 patients (median [IQR] age 59 [51-67] years; 189 female patients [58.0%]) of whom 11 were Asian or Mideast Indian (3.4%), 46 were Black or African American (14.1%), 5 were Hispanic or Latino (1.5%), 2 were Native Hawaiian or Other Pacific Islander (0.6%), 243 were non-Hispanic White (74.5%), 13 were more than 1 race (14.0%), and 6 declined to answer (1.9%) (Figure 1). Appendiceal tumors (115 patients [35.3%]) and colorectal tumors (86 patients [26.4%]) were the most common primary tumor types. Most patients received curative-intent treatment (258 patients [79.1%]) and had a preoperative American Society of Anesthesiology score of 3 or greater (258 patients [79.1%]). Cytoreductive surgery with or without hyperthermic intraperitoneal chemotherapy (241 patients [73.9%]) was the most performed procedure (eTable 1 in Supplement 1).

### AD Designation and Documentation Rates

**Figure 2.**
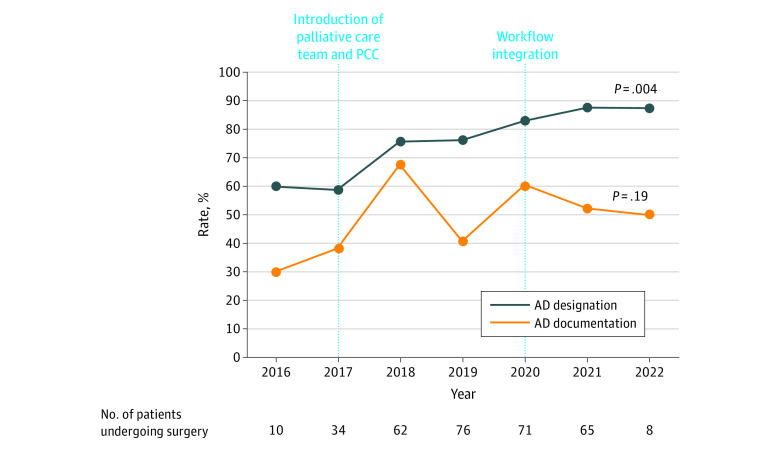
Advance Directive Designation and Documentation Rate Per Year Surgical case volumes in the respective years are represented in the bottom row. AD indicates advance directive; PCC, patient care coordinator.

Overall, 254 patients (77.9%) designated ADs, of which 170 ADs (52.1%) had AD documentation. The designation rate increased from 72.0% (131 of 182 patients) to 85.4% (123 of 144 patients) after workflow integration (*P* = .004) (Figure 2). Two prominent elevations in the AD designation rate were noted: 1 in 2018, which coincided with the onboarding of our palliative care team, and the other in 2020, which coincided with our workflow integration initiative. The AD documentation rate did not increase significantly after workflow integration (48.9% [89 of 182] ADs documented vs 56.3% [81 of 144] ADs documented; *P* = .19). Among patients with an AD designation, 248 (97.6%) had an HCPOA and 71 (27.9%) had an LW. Patients under 50 years of age were more likely to have an HCPOA (51 of 83 patients [61.4%]) than a LW (7 of 83 patients [8.4%]; *P* < .001).

Approximately one-half of the patients (157 patients [48.2%]) received palliative care consultation, most of whom had designated ADs (155 of 157 patients [98.7%]). The 2 patients without an AD designation underwent their operation before 2020 and did not wish to designate AD at the time despite evidence of discussion in palliative care clinic notes. The proportion of patients receiving palliative care consultation increased after workflow integration (77 of 182 patients [42.3%] vs 80 of 144 patients [55.6%], *P* = .02), aligning closely with the temporal increase in the AD designation rate. Notably, the AD designation rate also increased among patients who did not receive palliative care, although this was not statistically significant (56 of 105 patients [53.3%] before 2020 vs 19 of 31 patients [61.3%] in 2020 vs 24 of 33 patients [72.7%] in 2021-2022; *P* = .14).

### Timing of AD Designation With Respect to Surgery

Between 2016 and 2019, the earliest record of AD designation was made at a median (IQR) of 7 days (24 days preoperatively to 412 days postoperatively) after the index surgery due to documentation timelines. After workflow integration in 2020, this gap was reduced to a median (IQR) of 3 days (62 days preoperatively to 14 days postoperatively) after surgery Overall, 32.5% of patients (106 of 326 patients) had AD designation prior to surgery and this proportion increased after workflow integration (50 of 182 patients [27.5%] vs 56 of 144 patients [38.9%]; *P* = .03).

### Factors Associated With AD Designation and Documentation

In multivariable analyses, workflow integration was associated with increased odds of AD designation (odds ratio [OR], 2.05; 95% CI, 1.01-4.18; *P* = .048). A palliative care encounter was the most strongly associated factor with AD designation (OR, 41.48; 95% CI, 9.59-179.43; P <.001) and documentation (OR, 4.17; 95% CI, 2.57- 6.77; P < .001), followed by the highest age quartile for AD designation (OR, 3.79; 95% CI, 1.32-10.89; P = .01) and the highest age quartile for AD documentation (OR, 2.41; 95% CI, 1.21-4.79; P = .01) (Table 1 and Table 2). Palliative-intent treatment was associated with an increased odds of AD designation (OR, 5.12; 95% CI, 1.32-19.89; *P* =.02). In contrast, patients who self-identified as a race or ethnicity other than non-Hispanic White were less likely to have designated ADs (OR, 0.36; 95% CI, 0.17-0.76; *P* = .008).

**Table 1.** Demographic and Clinical Factors Associated With Advance Directive Designation

Factor	Univariable OR (95% CI)	*P* value	Multivariable OR (95% CI)	*P* value
Age, y (quartiles)				
≤50	1 [Reference]	NA	1 [Reference]	NA
51-59	2.00 (1.02-3.90)	.04	2.19 (0.92-5.25)	.08
60-66	2.89 (1.37-6.08)	.005	2.43 (0.97-6.06)	.06
≥67	5.37 (2.28-12.62)	<.001	3.79 (1.32-10.89)	.01
Sex				
Female	1 [Reference]	NA	NA	NA
Male	1.29 (0.76-2.22)	.35	NA	NA
Race and ethnicity				
Non-Hispanic White	1 [Reference]	NA	1 [Reference]	NA
Other^a^	0.43 (0.20-0.77)	.004	0.36 (0.17-0.76)	.008
Primary tumor site				
Appendix	1 [Reference]	NA	NA	NA
Colorectal	1.97 (0.91-4.23)	.08	NA	NA
Other abdominal	0.827 (0.45-1.52)	.54	NA	NA
Cutaneous melanoma	0.29 (0.09-0.97)	.04	NA	NA
Intent of treatment				
Curative	1 [Reference]	NA	1 [Reference]	NA
Palliative	9.46 (2.98-30.99)	<.001	5.12 (1.32-19.89)	.02
Palliative care encounter				
No	1 [Reference]	NA	1 [Reference]	NA
Yes	54.80 (13.10-228.53)	<.001	41.48 (9.59-179.43)	<.001
American Society of Anesthesiology score				
≤2	1 [Reference]	NA	1 [Reference]	NA
≥3	3.41 (1.90-6.09)	<.001	1.92 (0.90-2.08)	.09
Type of surgery performed				
Cytoreductive surgery	0.71 (0.20-2.49)	.59	NA	NA
Diagnostic	1 [Reference]	NA	NA	NA
Other therapeutic procedure	0.24 (0.06-0.90)	.03	NA	NA
Workflow integration				
Before	1 [Reference]	NA	1 [Reference]	NA
After	2.28 (1.30-4.01)	.004	2.05 (1.01-4.18)	.048

Abbreviations: NA, not applicable; OR, odds ratio.

^a^ Other included Asian and Mideast Indian, Black or African American, Hispanic or Latino, Native Hawaiian or Other Pacific Islander, and more than 1 race. Six patients declined to self-identify race or ethnicity and were excluded from regression analyses.

**Table 2.** Demographic and Clinical Factors Associated With Advance Directive Documentation

Factor	Univariable OR (95% CI)	*P* value	Multivariable OR (95% CI)	*P* value
Age, y (quartiles)				
≤50	1 [Reference]		1 [Reference]	NA
51-59	1.71 (0.93-3.14)	.09	1.68 (0.87-3.24)	.12
60-66	1.87 (0.99-3.51)	.05	1.58 (0.81-3.11)	.18
≥67	2.96 (1.57-5.60)	<.001	2.41 (1.21-4.79)	.01
Sex				
Female	1 [Reference]	NA	NA	NA
Male	1.17 (0.75-1.81)	.50	NA	NA
Race and ethnicity				
Non-Hispanic White	1 [Reference]	NA	1 [Reference]	NA
Other^a^	0.57 (0.34-0.96)	.03	0.59 (0.34-1.04)	.07
Primary tumor site				
Appendix	1 [Reference]	NA	NA	NA
Colorectal	1.10 (0.63-1.93)	.74	NA	NA
Other abdominal	0.90 (0.54-1.52)	.70	NA	NA
Cutaneous melanoma	0.29 (0.08-1.13)	.07	NA	NA
Intent of treatment				
Curative	1 [Reference]	NA	NA	NA
Palliative	1.61 (0.95-2.70)	.08	NA	NA
Palliative care encounter				
No	1 [Reference]	NA	1 [Reference]	NA
Yes	4.50 (2.82-7.12)	<.001	4.17 (2.57-6.77)	<.001
American Society of Anesthesiology score				
≤2	1 [Reference]	NA	NA	NA
≥3	1.62 (0.95-2.78)	.08	NA	NA
Type of surgery performed				
Cytoreductive surgery	1.21 (0.51-2.90)	.67	NA	NA
Diagnostic	1 [Reference]	NA	NA	NA
Other therapeutic procedure	0.75 (0.28-1.99)	.56	NA	NA
Workflow integration				
Before	1 [Reference]	NA	NA	NA
After	1.34 (0.87-2.08)	.19	1.03 (0.64-1.68)	.89

Abbreviations: NA, not applicable; OR, odds ratio.

^a^ Other included Asian and Mideast-Indian, Black or African American, Hispanic or Latino, Native Hawaiian or Other Pacific Islander, and more than 1 race. Six patients preferred not to disclose their race and were excluded from regression analyses.

### Overall Survival

**Figure 3.**
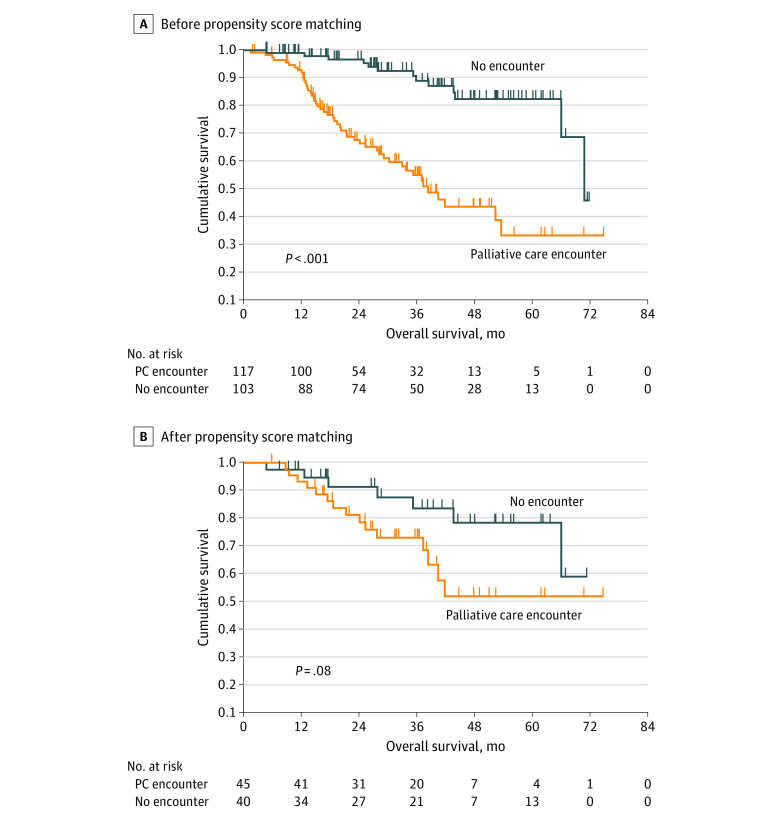
Comparison of Overall Survival Based on the Receipt of Palliative Care Consultation The figure shows the comparison of overall survival with receipt of palliative care among patients with a primary diagnosis of appendiceal cancer, colorectal cancer, and mesothelioma before propensity score match (A) and after propensity score matching (B). PC indicates palliative care.

Median (IQR) follow-up duration was 27 (15-43) months. In the cohort of patients who underwent therapeutic procedures for appendiceal neoplasms, colorectal neoplasms, and mesothelioma, those who had consulted with the palliative care team had shorter OS compared with those who had not (median OS, 38.3 months [95% CI, 31.1-45.5 months] vs 70.8 months [95% CI, X-Y] months; *P* < .001) (Figure 3A). However, after propensity score matching and selection of patients with a high probability (>0.5) of receiving palliative consultation, no significant difference was observed (median not reached in both groups) (Figure 3B and eTable 2 in Supplement 2).

## Discussion

This cohort study described an initiative targeting the systematic integration of ACP discussions at multiple levels of care for patients with advanced malignancies. Overall, the rates of AD designation (85.4%) and documentation (56.3%) in our patient cohort after workflow integration were markedly higher than those previously reported in oncology patients, high-risk surgical patients, and the general population outside an end-of-life setting (26%-37%).^10,11,12,38,39^ To our knowledge, this is the first initiative to establish a novel cohabiting unit of surgical and palliative care teams focused on improving the ACP process.^9,40^

Whereas medical and surgical oncologists are expected to address basic symptoms and initiate ACP discussions, palliative care specialists may be better suited to provide higher-order supportive care and aid in complex decision-making regarding ACP for patients with cancer.^41^ Prior recommendations encourage recurring interventions engaging multiple clinicians for effective ACP, leading to improved AD completion.^42,43^ Through our initiative, we leveraged patient communication throughout the multidisciplinary care team at multiple levels. We provided evidence that a palliative care encounter was associated with AD designation and documentation, possibly reflecting the influence of a dedicated ACP discussion recording section in our palliative care team’s EHR note template. Increasing proportions of patients visiting palliative care after workflow integration may partly account for this association; however, workflow integration was associated with improved AD designation irrespective of palliative care consultation, pointing toward the potential contributions of the other components of our initiative.

In our study, the first record of ADs in patients’ EHRs was within a week of their index surgery, indicating that the perioperative period is critical for engaging stakeholders in ACP discussions.^44^ The lag time between surgery and AD availability in the EHR decreased after workflow integration, and more patients recorded ADs preoperatively, which may potentially be related to the multiple levels of workflow integration. In our cohort, the preoperative AD designation rate after workflow integration (38.9%) was higher than the historically reported rates in high-risk surgical populations (15%-34%).^10,11,12,45^

In this study, we highlighted several established features regarding AD designation. More patients designated an HCPOA than an LW, a more daunting AD given the immediate emphasis it places on end-of-life care.^46^ Younger patients were less likely to have designated ADs, especially an LW. These observations are in line with the findings of Berkowitz and colleagues,^47^ whic attribute lower AD designation rates in younger populations to the lack of health care contact beyond their oncologist, compared with older patients who may have outpatient geriatrics, primary care, and palliative care contact. Patients who received palliative-intent treatment had greater odds of AD designation in multivariable analysis, possibly reflecting greater awareness of disease prognosis and the strong emphasis on goals of care by treating physcians.^48^ Importantly, patients self-identifying as a race or ethnicity other than non-Hispanic White had lower odds of AD designation, possibly reflecting disparities in socioeconomic status, health literacy, and cultural beliefs regarding the acceptability of ACP discussions.^12,49,50,51,52,53^

The association of early ACP with OS is unclear, as demonstrated in a systematic review^54^ of 43 randomized clinical trials regarding ACP interventions, 7 of which were pooled in a meta-analysis of survival outcomes (HR, 0.90; 95% CI, 0.69-1.17). Studies^35,36,55^ reporting reduced mortality postulate enhancements in quality of life and psychosocial support, reduction of symptom burden, and less aggressive end-of-life treatments as mechanisms for the potential survival benefit. In our cohort, patients with the highest symptom burden and pressing needs for end-of-life care discussion were seen by the palliative care team actively. Despite this bias, no significant survival differences were observed when compared with a propensity score-matched group of patients who were not consulted by the palliative care team. The importance of this finding is 2-fold. First, it emphasizes that palliative care referral is not inherently associated with worse survival, contradictory to negative perceptions of palliative care as a last resort in terminal stages, which is a common barrier to the early clinical integration of ACP.^54,56,57,58^ Second, these observations reveal a shortcoming of our initiative by highlighting an at-risk group of patients who did not receive palliative care consultations but may have benefited from them.

Our study also highlights systemic barriers to ACP delivery. The presence of suboptimal EHR documentation, discrepancies in the location of ADs, and potential delays between ACP discussions and EHR documentation indicate a lack of standardized processes for recording ACP information.^21^ This heterogeneity can impede clinicians from promptly accessing up-to-date directives in emergency situations and result in the loss of ACP-related information during care transitions across health networks. Therefore, there is a clear need for centralized and standardized ACP recording.^20^ Standardizing ACP billing codes and incorporating consistent documentation as a crucial benchmark for cancer center accreditation can help achieve this goal. The success of notable systemic ACP interventions supports systematic integration of ACP into specialty care and delivery models.^33,40,59^ Our model offers a means to effectively utilize existing palliative care resources while also holding the potential for external dissemination, particularly considering the nationwide expansion of palliative and supportive care facilities promoted by the Commission on Cancer accreditation standards.^60,61^

### Strengths and Limitations

A key strength of our study lies in the preoperative integration of palliative care in a largely curative oncologic setting, differentiating it from others applied primarily postoperatively and/or at the time of terminal diagnosis.^9,62,63,64^ Supporting clinicians in a cohabiting surgical care environment by utilizing existing systems was crucial to controlling institutional expenses associated with model design and delivery.^65^ In an increasingly cost-inaccessible system, billable ACP discussion time might add to up-front costs.^66,67^ However, evidence suggests that early palliative care integration may offset initial costs by optimizing resource utilization during end-of-life care, further supporting our model’s rationale.^5,26^

This study also has limitations. Our institution has an underlying infrastructure to facilitate the integrated model, limiting generalizability across care networks. Additionally, we may have underestimated the designation and documentation rates by not recognizing potential patients with physical AD copies that were not scanned into our EHR. Although ACP is a dynamic process, we evaluated static yet important benchmarks of the quality of palliative care delivery.^6,14,34,68^ Assessing qualitative outcomes, including the content of ACP discussions, postoperative quality of life, and the congruence of patient wishes with end-of-life care, was beyond the scope of this retrospective analysis and is important in future modeling.^10,69,70^ In line with previous reports,^51^ most patients in our cohort were non-Hispanic White, and AD designation rates were lower among other racial and ethnic groups. Future efforts in this regard may explore barriers unique to racial and ethnic minority groups and tailor interventions to provide culturally informed and competent care.^71^ We must also acknowledge that workflow integration in 2020 coincided with the COVID-19 pandemic, and we cannot ascertain the extent to which this may have contributed to the temporal increase in AD designation.^72,73^

## Conclusions

Our integrated ACP initiative was associated with increased AD designation rates among patients who underwent predominantly curative-intent surgical procedures for locoregionally advanced abdominal and soft tissue tumors at an academic center. The increase in AD designation and the overall rate of AD documentation were associated with an increase in the proportion of patients consulting palliative care, although additional components of the integrated workflow system likely contributed cumulatively. Most patients designated ADs near the time of surgery, indicating that the perioperative period was critical in engaging stakeholders in ACP discussions, and workflow integration was associated with earlier recording of ADs with respect to the index surgical event. Older age and palliative intent treatment were independently associated with AD designation, whereas patients who self-identified as races and ethnicities other than non-Hispanic White were less likely to have designated ADs. After propensity score matching, no significant differences in OS were observed on the basis of palliative care consultation. This service-level initiative, utilizing existing resources, highlights the benefits of early integrated palliative care as an essential component of multimodal oncologic care for complex patients undergoing surgery.^25^

## Notes

Supplement 1. **eAppendix 1.** Palliative Care Team EHR Clinic Note Template Summary With a Dedicated ACP Section Recording Patient Preferences for Advance Directives **eFigure.** New Patient Workflow Through the Regional Therapies Surgical Oncology Clinic After Workflow Integration **eAppendix 2**. Key Terms Used to Search for the Presence of ADs in Progress Notes **eTable 1.** Distribution of Baseline Variables Amongst Patients With and Without Advance Directive Designation **eTable 2.** Propensity Score Matching Characteristics: Comparison of the Cohorts Based on Variables Against Which Matching Was Performed

Supplement 2.
Data Sharing Statement
