# Supplementary material for: Early Integrated Palliative Care Within a Surgical Oncology Clinic
Source: JAMA Netw Open. 2023 Nov 7;6(11):e2341928. doi: 10.1001/jamanetworkopen.2023.41928 (PMC10630898; doi:10.1001/jamanetworkopen.2023.41928)
Supplement: Supplement 1. — eAppendix 1. Palliative Care Team EHR Clinic Note Template Summary With a Dedicated ACP Section Recording Patient Preferences for Advance Directives eFigure. New Patient Workflow Through the Regional Therapies Surgical Oncology Clinic After Workflow Integration eAppendix 2. Key Terms Used to Search for the Presence of ADs in Progress Notes eTable 1. Distribution of Baseline Variables Amongst Patients With and Without Advance Directive Designation eTable 2. Propensity Score Matching Characteristics: Comparison of the Cohorts Based on Variables Against Which Matching Was Performed [file jamanetwopen-e2341928-s001.pdf]

Supplemental Online Content

Bansal VV, Kim D, Reddy B, et al. Early integrated palliative care within a surgical oncology clinic. *JAMA Netw Open.* 2023;6(11):e2341928. doi:10.1001/jamanetworkopen.2023.41928

eAppendix 1. Palliative Care Team EHR Clinic Note Template Summary With a Dedicated ACP Section Recording Patient Preferences for Advance Directives

eFigure. New Patient Workflow Through the Regional Therapies Surgical Oncology Clinic After Workflow Integration

eAppendix 2. Key Terms Used to Search for the Presence of ADs in Progress Notes

eTable 1. Distribution of Baseline Variables Amongst Patients With and Without Advance Directive Designation

eTable 2. Propensity Score Matching Characteristics: Comparison of the Cohorts Based on Variables Against Which Matching Was Performed

This supplemental material has been provided by the authors to give readers additional information about their work.

© 2023 Bansal VV et al. JAMA Network Open.

eAppendix 1 - Palliative care team EHR clinic note template summary with a dedicated ACP section recording patient preferences for Advance Directives

ASSESSMENT & PLAN

[Name] is a [age] years old [sex] with diagnosis of ____ [(example: metastatic cecal mucinous adenocarcinoma with peritoneal carcinomatosis) (time of diagnosis)]. [Treatment (example: FOLFOX and Bevacizumab - last dose (date)) and ____ surgery with Dr. ____ on [date]. The patient had a remarkable medical history _____.

#Goals of Care:

- We discussed that it will be important to have ongoing discussions about his quality of life in the setting of his treatment.

#**Advance Care Planning**:

- Designated Power of Attorney: ____ [Name and relation to the patient]

- Advance Directives: I spoke to the patient about filling out an advance directive. The Health Care Power of Attorney form (from IDPH website Power of Attorney for Health Care (http://dph.illinois.gov/sites/default/files/forms/forms-legal-power-attorney-040716.pdf)) was given to the patient.

ATTENDING PHYSICIAN ATTESTATION

#Risk Assessment:

#Followup:

- The patient was advised to follow up with the palliative medicine clinic at the time of follow-up with Dr. ____ in the Surgical Oncology Clinic.

© 2023 Bansal VV et al. JAMA Network Open.

eFigure - New patient workflow through the Regional Therapies Surgical Oncology Clinic after workflow integration.

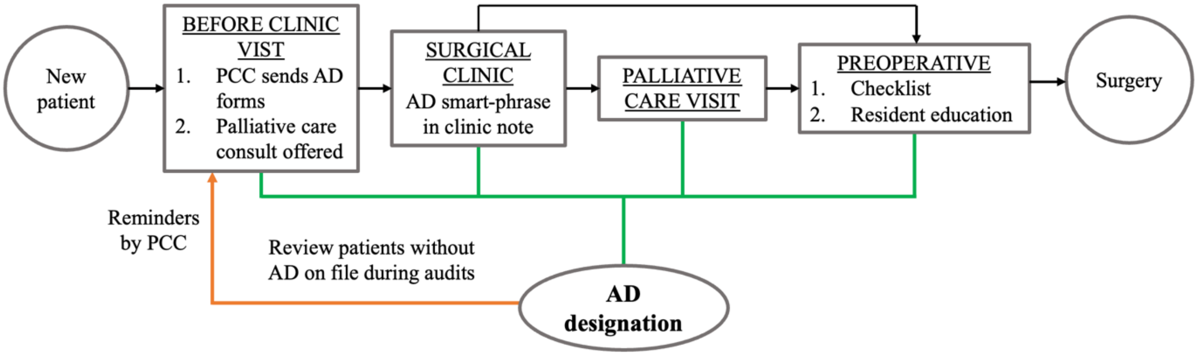

Abbreviations: PCC - Patient Care Coordinator; AD - Advance Directive

eAppendix 2 - Key terms used to search for the presence of ADs in progress notes

1. Power of Attorney-related
    - Power of Attorney
    - POA
    - DPOA
    - HCPOA
    - Surrogate
    - Proxy
2. Living Will-related
    - Living Will
    - LW
3. Physician Orders of Life-Sustaining Treatment (POLST)-related
    - POLST
4. Others
    - Goals of care
    - Palliat*

© 2023 Bansal VV et al. JAMA Network Open.

eTable 1: Distribution of baseline variables amongst patients with and without advance directive designation

	AD designation (n = 254)	No designation (n = 72)	p-value
**Age (quartiles)**			<0.001
≤ 50 years	52 (62.7%)	31 (37.3%)	
51-59 years	67 (77.0%)	20 (23.0%)	
60-66 years	63 (82.9%)	13 (17.1%)	
≥ 67 years	72 (90.0%)	8 (10.0%)	
**Sex**			0.347
Female	143 (76.1%)	45 (23.9%)	
Male	111 (80.4%)	27 (19.6%)	
**Race**			0.015
Non-Hispanic White	199 (81.9%	44 (18.1%)	
Other populations	51 (66.2%)	26 (33.8%)	
**Primary site**			0.034
Appendix	90 (78.3%)	26 (22.4%)	
Colorectal	75 (87.2%)	11 (12.8%)	
Mesothelioma	28 (77.8%)	7 (20.0%)	
Other abdominal	55 (85.7%)	22 (14.3%)	
Cutaneous Melanoma	6 (50.0%)	6 (50.0%)	
**Intent of treatment**			
Palliative	74 (96.1%)	3 (3.9%)	
Curative	180 (72.3%)	69 (27.7%)	
**Palliative care encounter**			<0.001
No	99 (58.6%)	70 (41.4%)	
Yes	155 (98.7%)	2 (1.3%)	
**ASA score**			<0.001
≤ 2	40 (58.8%)	28 (41.2%)	
≥ 3	214 (82.9%)	44 (17.1%)	
**Type of surgery performed**			<0.001
CRS	197 (81.7%)	44 (18.3%)	
Diagnostic	19 (86.4%)	3 (13.6%)	
Other therapeutic procedures	38 (60.3%)	25 (39.7%)	
**Year of Surgery**			0.004
2016-19 (Before workflow integration	131 (72.0%)	51 (28.0%)	
2020-22 (After workflow integration	123 (85.4%)	21 (14.6%)	

© 2023 Bansal VV et al. JAMA Network Open.

Footnotes:

Percentages represent row-wise proportions.

Other populations included Asian/Mideast-Indian (n = 11, 3.4%), Black/African-American (n = 46, 14.1%), Hispanic/Latino (n = 5, 1.5%), Native Hawaiian/Other Pacific-Islander (n = 2, 0.6%), more than one race (n = 13, 4.0%). Six (1.9%) patients declined to self-identify race.

Abbreviations: AD – Advance Directive, ASA – American Society of Anesthesiology, CRS – Cytoreductive Surgery

eTable 2 - Propensity score matching characteristics: Comparison of the cohorts based on variables against which matching was performed.

	**Palliative care encounter (n = 80)**	**No encounter (n = 80)**	**p-value**
Age (median, IQR)	57 (48.5-65.8)	59 (52.0-65.8)	0.657
ASA score ≥ 3	66 (82.5%)	66 (82.5%)	1
Curative treatment intent	72 (90.0%)	72 (90.0%)	1
Primary			0.84
Appendiceal	36 (45.0%)	35 (43.8%)	
Colorectal	32 (49.0%)	30 (37.5%)	
Mesothelioma	12 (15.0%)	15 (18.8%)	

© 2023 Bansal VV et al. JAMA Network Open.
