# Supplementary material for: Early Integrated Palliative Care Within a Surgical Oncology Clinic
Source: JAMA Netw Open. 2023 Nov 7;6(11):e2341928. doi: 10.1001/jamanetworkopen.2023.41928 (PMC10630898; doi:10.1001/jamanetworkopen.2023.41928)
Supplement: Supplement 2. — Data Sharing Statement [file jamanetwopen-e2341928-s002.pdf]

Data Sharing Statement

Bansal. Early Integrated Palliative Care Within a Surgical Oncology Clinic. *JAMA Netw Open*. Published November 07, 2023. doi:10.1001/jamanetworkopen.2023.41928

Data
Data available: No

Additional Information
Explanation for why data not available: The datasets generated and analyzed during the current study are not publicly available due to institutional regulations. Clinical data from this study may be made available upon reasonable request from qualified medical or scientific professionals, provided that the request aligns with the specified purpose and may involve de-identified individual participant data. Access to the requested data is granted after signing a data-access agreement.
